# Hypertrophic cardiomyopathy in young Maine Coon cats caused by the p.A31P cMyBP-C mutation - the clinical significance of having the mutation

**DOI:** 10.1186/1751-0147-53-7

**Published:** 2011-02-09

**Authors:** Mia TN Godiksen, Sara Granstrøm, Jørgen Koch, Michael Christiansen

**Affiliations:** 1 Department of Clinical Biochemistry and Immunology, Statens Serum Institut, Artillerivej 5, DK-2300 Copenhagen, Denmark; 2 Department of Small Animals Clinical Sciences, Faculty of Life Science, University of Copenhagen, Dyrlægevej 46, DK-1870 Frederiksberg C, Denmark

## Abstract

**Background:**

In Maine Coon (MC) cats the c.91G > C mutation in the gene *MYBPC3*, coding for cardiac myosin binding protein C (cMyBP-C), is associated with feline hypertrophic cardiomyopathy (fHCM). The mutation causes a substitution of an alanine for a proline at residue 31 (p.A31P) of cMyBP-C. The pattern of inheritance has been considered autosomal dominant based on a single pedigree. However, larger studies are needed to establish the significance of cats being heterozygous or homozygous for the mutation with respect to echocardiographic indices and the probability of developing fHCM. The objective of the present study was to establish the clinical significance of being homozygous or heterozygous for the p.A31P cMyBP-C mutation in young to middle-aged cats.

**Methods:**

The cohort consisted of 332 MC cats, 282 cats < 4 years (85%). All cats were examined by 2-D and M-mode echocardiography. DNA was extracted from blood samples or buccal swabs and screened for the p.A31P cMyBP-C mutation in exon 3 of the gene, using polymerase chain reaction followed by DNA sequencing.

**Results:**

The fHCM prevalence was 6.3% in the cohort. Eighteen cats were homozygous and 89 cats were heterozygous for the mutation. The odds ratio for having fHCM for homozygous cats was 21.6 (95% confidence interval 7.01-66.2) - when the group of equivocal cats was categorized as non-affected. Overall, 50% of the cats that were homozygous for the mutation had fHCM. p.A31P heterozygosity was not associated with a significant odds ratio for fHCM. In cats in the 4 to 6 years of age range a similar, non significant, odds ratio was seen in heterozygous cats. Only two cats over four years were homozygous and both were diagnosed with fHCM.

**Conclusion:**

As there is no significant odds ratio associated with being heterozygous for the pA31P cMyBP-C mutation at this age, the mutation must have a very low penetrance in this group. From our data it would appear that most MC cats that develop fHCM due to the p.A31P mutation prior to the age of approximately 6 years do so because they are homozygous for this mutation.

## Background

Hypertrophic cardiomyopathy (HCM), in humans, is a primary disorder of the myocardium that most commonly results from mutations in genes that encode for sarcomeric proteins. Feline HCM (fHCM) is a clinically heterogeneous disorder which is characterised by localized or generalized concentric left ventricular hypertrophy and diastolic dysfunction [1-7]. Affected cats may progress into congestive heart failure, thromboembolic events or sudden cardiac death [8]. Not much is known about the genetics underlying fHCM and presently only two mutations have been found [4,5].

The Maine Coon (MC) cat is predisposed to fHCM. The true prevalence within the breed is not known, however it may be as high as 9.5-26.3% [9]. Similar to human HCM, fHCM in MC cats exhibits incomplete penetrance and variable expressivity; thus, it is possible to find phenotypically normal mutations carriers [10,11]. Diagnosis of fHCM in MC cats should ideally be based on a positive family history, and a thorough echocardiographic assessment of several imaging planes of the heart with follow-ups. Genetic testing is currently of limited utility, as the clinical significance of being a mutation carrier has not been completely established. fHCM in MC cats is an excellent spontaneous animal model for human HCM, as the characteristics of the disease mimic the ones seen in human patients including the increased risk of sudden death [7,12].

Mutations in the *MYBPC3 *gene, encoding the sarcomere cardiac protein Myosin Binding Protein C (cMyBP-C), are associated with HCM in human and fHCM in MC and Ragdoll cats [4,5,13]. More than 240 HCM-causing mutations in the cMyBP-C protein have been reported from studies of human HCM [14], and cMyBP-C mutations are responsible for ~ 30% of all human HCM cases [3,13,15].

Meurs *et al*. [4] identified a disease-causing missense mutation (c.91G > C) in the feline *MYBPC3 *gene in a colony of MC cats with fHCM. The mutation causes the substitution of an alanine for a proline at residue 31 (p.A31P) of the cMyBP-C protein. Affected cats exhibit a broad phenotypic variation from mild to severe fHCM. Some cats have died before four years of age, where others were still alive at 8-12 years of age [4]. The frequency of this mutation has later been reported to be 34% among MC cats [16].

The objective of this study was to investigate the relationship between fHCM and MC cats heterozygous and homozygous for the p.A31P cMyBP-C mutation in a large cohort of MC cats. This study may contribute recommendations to MC breeding programs concerning the control of fHCM.

## Methods

### Clinical examinations

A cohort of 332 MC cats was prospectively included in the study at the Department of Small Animal Clinical Sciences, University of Copenhagen, Denmark. The cohort consisted of MC cats from MC breeders and owners who gave informed consent to participate. The study was approved by the ethics committee of the department. All cats were examined by 2-D and M-mode echocardiography in right lateral recumbency and imaged from below by one trained observer using a Vivid 7 Dimension ultrasonographic system equipped with a 10 S phased array transducer (4-11.5 MHz; GE Healthcare, Horten, Norway). Measurements of the left ventricle were obtained from M-mode imaging in standard echocardiographic right parasternal long axis four-chamber and short axis views at level of the chordae tendineae and according to the recommendations of the Echocardiography Committee of the Specialty of Cardiology, American College of Veterinary Internal Medicine and the American Society of Echocardiography, respectively [17,18]. The M-mode values of left ventricular dimensions were confirmed by measurements of multiple left ventricular wall segments from several 2-D views and cats were classified to have fHCM if the maximum diastolic wall thickness in any segment exceeded 5.5 mm in > 50% of segment length. Presence of an enlarged left atrium, systolic anterior motion of the septal leaflet of the mitral valve, left ventricular end-systolic cavity obliteration and enlarged papillary muscles further strengthened the diagnosis of fHCM. Cats were considered to be fHCM negative if the diastolic 'left ventricular free wall' (LVFW) and diastolic 'interventricular septum' (IVS) measured < 5 mm and no other cardiac abnormalities could be found. Cats were categorized as equivocal if they had a normal wall thickness (< 5.5 mm) and displayed papillary muscle hypertrophy.

An fHCM screening form, from PawPeds international health programme [19], was filled out immediately after the examination and all images were stored digitally for later off-line analysis. All values represented the average of three consecutive beats. The method used to measure left atrium and aorta has previously been described in dogs [20].

The p.A31P cMyBP-C genotype of all cats was unknown to the observer at the echocardiographic examination and later offline analysis.

### Laboratory studies

DNA was extracted from ethylenediaminetetraacetic acid stabilized blood or full blood using automated DNA purification by MAXWELL^® ^(Promega, Nacka, Sweden) according to manufacturer's instructions. Where blood was not available (3% of all samples), DNA was obtained and extracted using a MasterAmp™ Buccal Swab Kit (VWR & Bie & Berntsen, Herlev, Denmark) after the manufacturer's instructions. The feline *MYBPC3 *gene sequence was obtained from Ensembl (ENSFCAG00000002530) [21]. Amplification of genomic DNA was performed using the following primer set: exonic forward primer 5'-agccttcagcaagaagcca-3' and exonic reverse primer 5'-caaacttgaccttggaggagc-3'. The polymerase chain reaction (PCR) was carried out with 1 μl genomic DNA (~ 50 ng/μl) in a volume of 25 μl containing 1 μl 20 pmol/μl primer mix (DNA technology AS, Aarhus, Denmark) 2.5 μl 10 × PCR buffer (15 mM MgCl_2_) (Qiagen, Copenhagen, Denmark), 0.5 μl dNTP mix 10 mM solution (GE Healthcare Life Sciences, Brondby, Denmark), 0.2 μl Hot star polymerase (Qiagen, Copenhagen, Denmark) and 5 μl Q-buffer (Qiagen, Copenhagen, Denmark). Samples were heat activated at 95°C for 15 min followed by 35 cycles: 95°C for 30 sec, 58°C for 30 sec, 72°C for 1 min and a final step of elongation (72°C for 7 min). PCR products were visually verified and thereafter treated with Exonuclease I (Medinova Scientific, Glostrup, Denmark). Mutation screening was carried out by direct DNA sequencing using BigDye^® ^technology (GE Healthcare Life Sciences, Brondby, Denmark) on an ABI 3730 sequencer (Applied Biosystems, Naerum, Denmark). PCR products were sequence in both directions using the respective forward and reverse primers.

### Statistics

The Chi-square test (χ^2^) was used to evaluate if the genotype distribution was in Hardy-Weinberg equilibrium, a *P*-value < 0.05 indicated significance. The clinical significance of the p.A31P cMyBP-C protein mutation was determined by looking at the probability of developing fHCM when comparing the heterozygous and the homozygous mutation carriers with the wild type cats using odds ratio calculation and the 95% confidence interval (95% Cfi) was established.

When data were plotted, the measurements of age, weight, diastolic IVS and LVFW, systolic LVFW and the ratio of left atria over aorta (LA/Ao) all followed a non-normal distribution, thus Kruskal-Wallis test was used to compare the medians of the data. A *P-*value < 0.05 was considered statistically significant.

**Figure 1:**
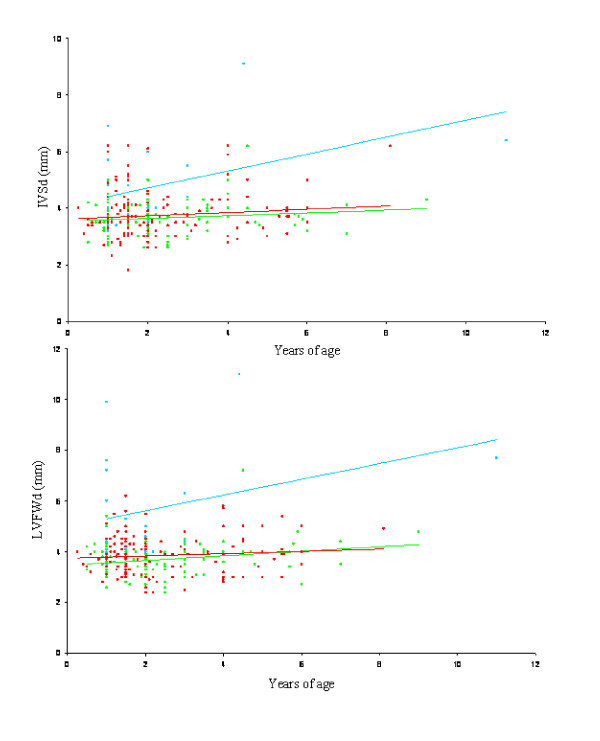
**The correlation with age and diastolic IVS and LVFW measurements as a function of c.91C *MYBPC3 *alleles**. Top: Plot of diastolic IVS correlation with age as a function numbers of c.91C *MYBPC3 *alleles. There was tendency to an increasing effect of p.A31P cMyBP-C homozygosity with age, though the tendency was not significant, *ρ *= 0.41, *P = 0.09*. There was only a low effect of being p.A31P cMyBP-C heterozygous, ρ = 0.09, *P *= 0.4. Bottom: Plot of diastolic LVFW correlation with age as a function of numbers of *MYBPC3 *c.91C alleles. The tendency was more weak than seen above, ρ = 0.2, *P *= 0.50. Red indicates the cMyBP-C wild type coordinates and the corresponding line of tendency. Green indicates p.A31P cMyBP-C heterozygous coordinates and the corresponding line of tendency. Blue indicates p.A31P cMyBP-C homozygous where and the corresponding line of tendency. Figure abbreviations: IVSd: diastolic interventricular septum, LVFWd: diastolic left ventricle free wall.

Spearman correlation was used to test if diastolic IVS and LVFW correlated with age for cats being homozygous for the mutation (Figure 1), a *P*-value < 0.05 was considered significant.

## Results

### The Maine Coon cohort

The MC cohort consisted of 332 cats, 118 males with a median age of 1.5 years of age (95% range 0.7; 6.0) and a median weight of 6.0 kg (95% range 4.0; 8.8) and 214 queens with a median age of 2.0 years (95% range 1.0; 5.5) and a median weight of 4.4 kg (95% range 3.4; 6.0). Presentation of the cohort can be seen in Tables 1 and 2, where the cats were categorized according to their fHCM clinical presentation and p.A31P cMyBP-C, respectively.

**Table 1:** Presentation of Main Coon cat cohort.

	fHCM status		
	fHCM	Equivocal	Negative
N, gender distribution	21 (14 males)	26 (14 males)	285 (90 males)
Age, y (median, 95% range)	2.0, 1.0; 11.0	2.0, 0.5; 5.5	2.0, 1.0; 5.3
Weight, kg (median, 95% range)	6.2, 4.5; 7.8	5.5, 3.5; 8.2	4.5, 3.4; 7.0
IVSd, mm (median, 95% range)	6.0, 3.4; 6.9	3.5, 2.7; 5.1	3.7, 2.8; 4.5
LVIDd, mm (median, 95% range)	15.0, 11.1; 19.0	15.5, 12.3; 19.0	15.7, 12.7; 19.0
LVFWd, mm (median, 95% range)	6.2, 4.9; 9.9	4.0, 2.8; 5.5	3.8, 2.8; 4.8
IVSs, mm (median, 95% range)	8.5, 5.8; 11.3	6.4, 4.5; 8.1	6.4, 4.8; 8.0
LVIDs, mm (median, 95% range)	7.8, 3.9; 11.3	8.2, 6.3; 13.0	9.1, 6.1; 12.5
LVFWs, mm (median, 95% range)	9.2, 6.8; 13.4	7.3, 6.0; 8.7	6.7, 5.0; 8.2
LA/Ao, (median, 95% range)	1.2, 1.0; 3.0	1.0, 1.0; 1.2	1.0, 0.9; 1.2
% with murmur	62	14	1
% with SAM	52	0	0
% with obliteration	43	43	< 1
% with hypertrophic papillary muscle	76	73	1
% with increased LA	14	4	< 0.5

IVSd: diastolic intraventricular septum, LVIDd: diastolic left ventricular inner diameter, LVFWd: diastolic left ventricular free wall, IVSs: systolic intraventricular septum, LVIDs: systolic left ventricular inner diameter, LVFWs: systolic left ventricular free wall, LA/Ao: left atrium over aorta.

**Table 2:** Presentation of the Main Coon cat cohort categorised in groups depending on the *A31P CMYBP-C *genotype.

	cMyBP-C p.A31P genotype			*P-*value mutation negative vs	
	Homozygous (C/C)	Heterozygous (G/C)	Wild type (G/G)	Homozygous	Heterozygous
n, gender distribution	18 (12 males)	89 (25 males)	225 (81 males)	-	-
Age, y (median, 95% range)	1.5, 1.0; 11.0	2.0, 0.9; 5.9	1.7, 1.0; 5.0	0.48	0.14
Weight, kg (median, 95% range)	5.9, 3.4; 7.0	4.6, 3.5; 7.8	4.5, 3.4; 7.2	0.04	0.32
IVSd, mm (median, 95% range)	4.2, 3.2; 9.1	3.7, 2.8; 5.5	3.7, 2.8; 5.1	0.002	0.70
LVIDd, mm (median, 95% range)	15.6, 10.2; 18.4	15.9, 12.8; 19.0	15.6, 12,3; 19.0	0.88	0.59
LVFWd, mm (median, 95% range)	5.1, 3.6; 11.0	3.7, 2.7; 4.8	4.0, 2.9; 5.0	< 0.001	0.10
IVSs, mm (median, 95% range)	7.8, 5.0; 11.3	6.3, 4.8; 8.1	6.4, 4.8; 8.4	0.04	0.29
LVIDs, mm (median, 95% range)	8.8, 4.0; 13.0	9.0, 5.7; 12.6	9.0, 6.1; 12.4	0.23	0.80
LVFWs, mm (median, 95% range)	8.9, 5.5; 13.4	6.7, 5.1; 8.3	6.7, 5.1; 8.7	0.016	0.90
LA/Ao, (median, 95% range)	1.1, 0.9; 4.0	1.0, 0.9; 1.1	1, 0.9; 1.2	0.0004	0.31
% with murmur	50	2	4		
% with SAM	44	1	0		
% with obliteration	44	4	5		
% with hypertrophic papillary muscle	56	9	8		
% with increased LA	22	0	< 1		

IVSd: diastolic intraventricular septum, LVIDd: diastolic left ventricular inner diameter, LVFWd: diastolic left ventricular free wall, IVSs: systolic intraventricular septum, LVIDs: systolic left ventricular inner diameter, LVFWs: systolic left ventricular free wall, LA/Ao: left atrium over aorta.

The cats were categorized into three groups: fHCM positive, fHCM negative and equivocal cats. The fHCM positive group consisted of 21 cats (14 males). The equivocal group consisted of 26 cats (14 males) and the remaining 285 cats were classified as fHCM negative (90 males). No significant differences in age was found between the three groups (*P *> 0.05). The weight of fHCM positive cats was significantly higher than fHCM negative cats (*P *< 0.05), no difference in weight was found between the fHCM cats and cats with equivocal status (*P *> 0.05).

### Characterisation of the fHCM positive MC cats

Twenty-one cats were diagnosed with fHCM, making the prevalence 6.3% - and 14.2% when the equivocal group was added to the fHCM affected group. However, for the male MC cats alone the fHCM prevalence was 11.9% and a further 11.9% were categorized as equivocal (a total of 23.7%). See Table 3 for genotype and phenotype distribution.

**Table 3:** Distribution of the fHCM negative, the equivocal and the fHCM positive cats in respect to p.A31P genotype.

	Negative	Equivocal	fHCM
Wild type (G/G)	200	16	10
Heterozygous (G/C)	78	8	2
Homozygous (C/C)	7	2	9

**Figure 2:**
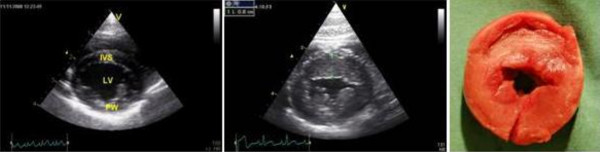
**2-D echocardiographic recordings of papillary muscles from a normal and an fHCM affected cat**. Left illustration: Right parasternal short axis of the left ventricle with papillary muscles view from a normal MC cat. Middle illustration: Right parasternal short axis of the left ventricle with papillary muscles view from a cat with severe hypertrophic cardiomyopathy. Right illustration: Gross heart specimen from a cat with severe concentric hypertrophy due to fHCM. Figure abbreviations: IVS: Intraventricular septum, LV: left ventricle and PW: posterior wall (free wall).

**Figure 3:**
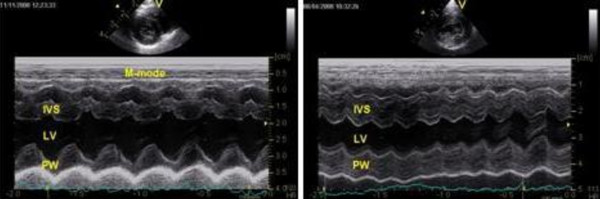
**Illustration of M-mode echocardiographic recordings from a normal and an fHCM affected cat**. Left illustration: M-mode echocardiogram recorded from an MC cat at the level of chordae tendinae shows normal left ventricular internal dimension, septum and posterior wall of the left ventricle. Right: M-mode echocardiogram from and an MC cat with severe fHCM with atrial fibrillation (HR 325/min), congestive heart failure and tromboembolic disease, recorded at the level of the chordae tendinae to assess left ventricular dimensions shows severe thickening of both the septum and posterior wall of the left ventricle. Figure abbreviations: IVS: Intraventricular septum, LV: left ventricle and PW: posterior wall.

In total, 13 fHCM positive cats (62%) had a cardiac murmur (3 with grade I, 4 with grade II, 5 with grade III, 1 with grade IV). Systolic anterior movement (SAM) of the mitral valve was observed in 52% of fHCM positive cats and end-systolic cavity obliteration was observed in 43% of fHCM positive cats. The diagnostic findings for the three groups are summarized in Table 1. The wall thickness and the inner diameter of the ventricle given in Tables 1, 2 and 4 reflect standard measurement with M-mode echocardiography from a right parasternal short axis view at level of the chordae tendinae. Localized and asymmetrical thickening of the myocardium can be missed with a standard M-mode projection in all cats, thus they were also measured by 2-D echocardiography. The echocardiographic measurements and M-mode echocardiograms of hearts from an fHCM positive and a negative MC, respectively, are shown in Figures 2 and 3.

**Table 4:** Echocardiographic characteristics for cats over 4 years of age.

	cMyBP-C p.A31P genotype		*P-*value
	Heterozygous (G/C)	Wild type (G/G)	
n, gender distribution	14 (4 males)	34 (14 males)	-
Age, y (median, 95% range)	5.7, 4.0; 9.0	4.5, 4.0; 6.0	0.17
Weight, kg (median, 95% range)	6.0, 4.5; 8.8	5.1, 8.7; 3.5	> 0.05
IVSd, mm (median, 95% range)	3.7, 3.1; 6.2	3.9, 2.9; 6.2	0.40
LVIDd, mm (median, 95% range)	16.6, 13.8; 19.3	15.9, 13.8; 1.9	0.20
LVFWd, mm (median, 95% range)	4.0, 2.7; 7.2	4.0, 2.9; 5.8	0.70
IVSs, mm (median, 95% range)	6.6, 5.0; 12.4	6.7, 5.0; 9.0	0.82
LVIDs, mm (median, 95% range)	9.6, 3.9; 12.9	10.0, 6.3; 14.0	0.83
LVFWs, mm (median, 95% range)	7.0, 5.2; 9.2	7.0, 4.1; 9.8	0.08
LA/Ao, (median, 95% range)	1.0, 0.9; 1.3	1.0, 0.9; 1.4	0.06
% with murmur	7	0	
% with SAM	7	0	
% with obliteration	14	3	
% with hypertrophic papillary muscle	7	12	
% with increased LA	0	0	

IVSd: diastolic intraventricular septum, LVIDd: diastolic left ventricular inner diameter, LVFWd: diastolic left ventricular free wall, IVSs: systolic intraventricular septum, LVIDs: systolic left ventricular inner diameter, LVFWs: systolic left ventricular free wall, LA/Ao: left atrium over aorta.

The equivocal cats were mainly characterized by normal wall thickness (< 5.5 mm) and papillary muscle size, with or without end-systolic cavity obliteration. In odds ratio calculations the equivocal cats were first added to the group of fHCM positive cats and afterward added to the group of fHCM negative cats for the same calculations.

### p.A31P cMyBP-C genotyping

**Figure 4:**
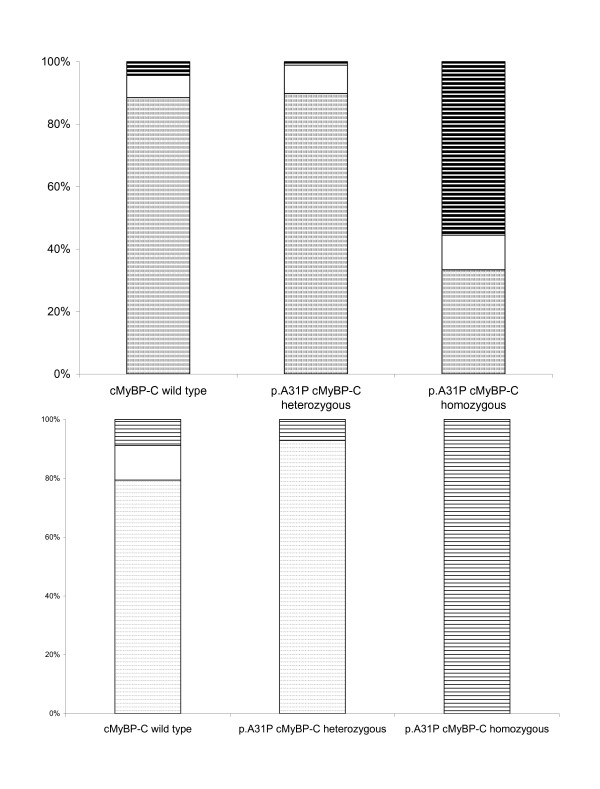
**Distributions of fHCM and equivocal cases in respect to p.A31P cMyBP-C genotype status**. A: Histogram of the distribution for the entire cohort. The group of p.A31P cMyBP-C homozygous cats had an increased proportion of fHCM cases. B. Histogram of the distribution for cats over 4 years of age. Except for the homozygous the distribution was very similar to the distribution shown in A. Parts of boxes marked with small dots represent fHCM negative cats. Blank parts of the boxes indicate equivocal cases. Box marked with black stripes indicate cases of fHCM.

All cats were genotyped with respect to the cMyBP-C mutation (Table 3). Eighteen MC cats were homozygous, 89 MC cats were heterozygous and 225 MC cats were wild type (no mutation). 10 out of 21 MC cats with an fHCM diagnosis did not carry the mutation. The histogram in Figure 4A shows the fraction of fHCM and equivocal cases in the three different genotypes.

The mutated *MYBPC3 *gene-allele (c.91C) was found to be the minor allele with an allele frequency of 0.19. The genotype distribution was not in Hardy-Weinberg equilibrium (*P <*0.05, χ^2 ^test).

### The significance of the p.A31P cMyBP-C mutation

Odds ratio calculations were used to determine the probability of developing fHCM in all cats with the mutation. The odds ratio was calculated for heterozygous and homozygous carriers of p.A31P cMyBP-C between fHCM affected and non-affected cats, using mutation wild type cats as a control group. The odds ratio of having fHCM (or being equivocal) for all cats carrying the C allele (c.91C) was found to be 1.9 (95% Cfi: 1.0; 3.6). The odds ratios of having fHCM for homozygous and heterozygous mutation carriers were 12.1 (95% Cfi: 4.3; 33.9) and 1.0 (95% Cfi: 0.5; 2.1), respectively. The same odds ratio calculations, with the equivocal cats added to the group of fHCM negative cats revealed an odds ratio for cats carrying the C allele (c.91C) to be 2.5 (95% Cfi: 1.0; 6.0). For homozygous and heterozygous cats the odds ratios were found to be 21.6 (95% Cfi: 7.0; 66.2) and 0.5 (95% Cfi: 0.1; 2.3), respectively.

The fHCM prevalence for the disease was found to be 2.4% in heterozygous cats and 9.1% of heterozygous cats were classified as equivocal. Among p.A31P cMyBP-C homozygous MC cats, 50% had fHCM and a further 11% were categorized as equivocal (Table 3). Table 5 gives an overview of the fHCM penetrance and the fHCM odds ratio of the group of heterozygous cats, the homozygous cats and both combined. The fHCM odds ratios of all three categories were compared to cMyBP-C wild type cats.

**Table 5:** The fHCM odds ratio and fHCM prevalence of the three genotype categories: heterozygous, homozygous and the hetero- and homozygous combined.

Genotype	fHCM prevalence	fHCM + equivocal prevalence	fHCM Odds ratio*	fHCM + equivocal odds ratio
All (G/G)+(G/C)+(C/C)	6.3%	14.2%	-	-
Heterozygous (G/C)	2.3%	12.8%	0.5 (95% Cfi: 0.1; 2.3)	0.8(95% Cfi: 0.3; 1.8)
Homozygous (C/C)	50%	61.1%	21.6 (95% Cfi: 7.0; 66.2)	12.1 (95% Cfi: 4.3; 33.9)
All carriers (G/C)+(C/C)	10.3%	19.8%	2.5 (95% Cfi: 1.0; 6.0)	2.9 (95% Cfi: 1.6; 5.5)

* The equivocal cats have been added to the non-affected cats

In all three categories the odds ratio was calculated as compared to the group of wild type cats.

The fHCM diagnostic parameters compared between groups of the three different genotypes are summarized in Table 2.

Figure 1 represents plots of the echocardiographic measurements of diastolic IVS and LVFW as a function of age and p.A31P cMyBP-C heterozygous or homozygous status. There was no significant correlation between age and diastolic IVS or LVFW in cats heterozygous for the mutation or in wild type cats. There was a tendency for diastolic IVS (ρ = 0.4, *P = *0.09) to increase with age for cats homozygous for the mutation.

A small cohort consisting of all cats over 4 years of age (with a median age of 4.9 years, 95% range 4; 8.1) was used to examine the significance of being affected by the p.A31P cMyBP-C mutation in middle-aged cats. The small cohort consisted of 50 cats; six of which had been positively diagnosed with fHCM. Within this cohort, we identified 14 cats being heterozygous and two cats being homozygous for the mutation. One equivocal cat and one fHCM positive cat were among the heterozygous cats and both homozygous cats were fHCM positive. The odds ratio of being fHCM positive for heterozygous mutation carriers over four years of age was found to be 0.64 (95% Cfi 0.1-3.6) - when the equivocal cats were categorized as fHCM affected. Categorizing the equivocal cats as non-affected resulted in an odds ratio of 0.8 (95%Cfi: 0.1-8.4). The relative distribution of fHCM and equivocal cats as a function of the p.A31P cMyBP-C genotype is shown in Figure 4B. Table 4 presents the echocardiographic measurements corresponding to either heterozygous or non-mutation carriers.

## Discussion

We investigated the clinical significance of being heterozygous or homozygous for the p.A31P cMyBP-C mutation in a cohort of young MC cats. We did not find a significant odds ratio for the development of fHCM for cats heterozygous for the mutation. However, we did find a significantly high odds ratio for cats homozygous for the mutation and so a high probability for developing fHCM. Based on these results the penetrance in heterozygous cats must be considered very low in young cats and high in young cats homozygous for the mutation.

Additionally, we found that diastolic IVS thickness had a tendency to progress with age in p.A31P cMyBP-C homozygous cats compared to heterozygous and wild type cats. A reduced number of homozygous cats due to fHCM related death might explain why the correlation between increasing age and increasing diastolic IVS or LVFW was not significant.

To compensate for the young mean age of the cohort we investigated a smaller group consisting of 50 cats, all over four years of age with a median of 4.9 years of age (95% range: 4; 8.1). In this small cohort, 14 cats were heterozygous and a further two were homozygous. Again, no significant association between p.A31P cMyBP-C and fHCM was found in heterozygous cats. This again indicated that p.A31P cMyBP-C associated fHCM was a disease with a very low penetrance also in middle-aged cats heterozygous for the mutation and highly penetrant for cats homozygous for the mutation. We propose that the p.A31P cMyBP-C mutation results in an inheritance patterns that resembles a recessive form of inheritance in young MC cats.

However, the clinical significance of the heterozygous mutation carrier status must be established in a cohort of older cats before conclusions regarding mode of inheritance are finalized and firm breeding recommendations made. Therefore, follow-up studies of the cohort are recommended with a delay of 2-5 years from this study to evaluate the clinical course of the heterozygous in respect to the probability of late-onset fHCM. Very recently, Wess *et al*. [11] stated that the p.A31P mutation is less pathogenic than reported so far based upon a cohort of 82 MC cats (mean age approx. 70-72 months). However, we find the number of homozygous cats in that study were too low (n = 3) to reliably assess the fHCM risk associated with this genotype [11]. The low number of homozygous cats might be the result of fHCM-associated yearly death. That study supports our results concerning heterozygous mutation carriers and fHCM development. However, another study has reported tissue Doppler imaging evidence of diastolic dysfunction in MC cats heterozygous for the mutation indicating that even though there may not be overt evidence of fHCM there is evidence of occult fHCM in these cats [10]. If occult disease is taken into consideration then an autosomal dominant mode of inheritance needs to be considered.

Recessive, or gene dosage dependent, mode of inheritance has previously been shown to be the case in some human patients with cMyBP-C associated HCM [22]. It can not be excluded that the p.A31P cMyBP-C mutation may exhibit a dominant pattern of inheritance pattern in a single colony of cats as previously described [4]. This may be a consequence of various factors, which may modify the phenotype. It is possible that those pedigrees with cats heterozygous for the p.A31P mutation and affected with fHCM may represent digenic inheritance or compound heterozygosity where another yet undiscovered mutation is present with the p.A31P cMyBP-C mutation. Digenic or compound heterozygosity is present in 8-30% of human HCM cases [13,15,23]. This suggests the need to conduct a mutation screening of all the HCM associated sarcomeric genes in MC cats.

Seven out of 21 MC cats with an fHCM diagnosis were not carrying the mutation, revealing that the etiology of fHCM is heterogeneous (i.e. at least one more cause is present) in the MC breed. Human HCM is associated with one or more mutations in at least 11 cardiac sarcomere genes [15]. In total, eight cardiac sarcomere genes associated with human HCM have been screened for fHCM disease-causing mutations in 14 cats from five different breeds [24]. The study did not identify any new disease-causing mutations in the sarcomere genes *MYH7*, *MYBPC3*, *TNNT2*, *TNNI3*, *TPM1*, *MYL2*, *MYL3 *and *ACTC*. However, these genes should still be considered as candidate genes for fHCM, as the cohort used in the study is very small [24]. By analogy to human HCM, it would seem very unlikely to identify mutations in these genes in a small patient group as the genes are - individually - rarely affected [13,15].

Defining a "normal heart" is a complicated clinical task that involves serial measurements and collecting a sufficient variety of pre-clinical and clinical information. Each specific heart disease, in principle, needs a specific reference interval or decision limits (cut-off points). Decision limits for fHCM with diastolic LVFW and IVS > 6 mm may impose difficulties in screening for fHCM in phenotypically normal "carriers" or in cats with mutations that cause late-onset fHCM. In early stages of disease, a high percentage of false negative results may be the consequence of too high a cut-off point.

The group of equivocal MC cats may contain a high number of both "false positive" and "false negative" cats. The category contains cats with normal wall thickness (< 5.5 mm) and papillary muscle hypertrophy, with or without obliteration. Consequently, the term "equivocal" was given to cats in which the heart showed some of the echocardiographic signs suggestive of fHCM, but without being distinctive enough to classify the cat as fHCM positive. These cats were in a grey zone and follow-up echocardiographic examination was always recommended to this group.

The present reference interval was established in a homogeneous MC population at large. No "normal" female or male cats had wall thickness > 5 mm and only three out of twenty-six equivocal cats were in the range from 5-5.5 mm. Although a lowering of a decision limit from 6 to 5.5 mm may cause slightly higher false positive results, we found that a 5.5 mm upper limit was a more appropriate value for screening for fHCM. This was a finding in agreement with a previous study where 5.0 mm is suggested as normal upper limit for myocardial thickness in MC [9].

The p.A31P cMyBP-C genotype distribution was not in Hardy-Weinberg equilibrium, indicating that there was a bias of selection in the MC cohort. In addition, the dis-equilibrium could possibly be explained by a reduced number of homozygous cats, which supports that the p.A31P cMyBP-C mutation was disease-causing and resulted in an increased mortality even in young cats. This is comparable with the findings in human HCM, where an early clinical debut of HCM is associated with a very poor prognosis [25]. Breeders volunteered their cats to be enrolled in the study, thus breeders with no interest in fHCM and fHCM genetics were not likely to participate. Furthermore, MC cats from breeding programs are bred based on certain selection criteria. Breeders with a commercial interest only use the strongest male and female cats for reproduction. That the prevalence of fHCM and of the p.A31P cMyBP-C mutation found in our cohort is similar to previously reported prevalence reduces bias though [9,16,26].Finally, only one-third of the cats in our cohort were males, this bias may result in underestimation of the fHCM prevalence, as fHCM is more common in male cats.

## Conclusions

In conclusion, p.A31P cMyBP-C associated fHCM is a disease with very low penetrance in young heterozygous cats. Our results support the pathogenic role of p.A31P when two affected gene alleles are present in a MC cat. Homozygosity of the cMyBP-C mutation only explained 43% of fHCM cases in the MC cohort therefore we recommend further large-scale genetic studies to identify potential disease-causing mutations in genes including the sarcomere genes most commonly involved in human HCM. Furthermore, due to the high probability of developing fHCM in the p.A31P cMyBP-C homozygous cats the 'production' of homozygous MC cats should be avoided. Thus, although genotyping of the p.A31P cMyBP-C mutation can not stand alone in limiting fHCM in MC, it is very important that breeders are aware of the genotype status and breeders should be informed of breeding recommendations. Breeding recommendations concerning this genetic variant are still controversial.

## List of abbreviations

c.91G > C: guanine substituted for a cytosine at the 91^th ^nucleotide of the coding gene sequence; Cfi: confidence interval; cMyBP-C: cardiac myosin binding protein C; fHCM: feline HCM; HCM: Hypertrophic cardiomyopathy; IVS: interventricular septum; LA/Ao: ratio of left atria diameter over aorta; LV: left ventricle; LVFW: left ventricular free wall; LVID: left ventricular inner diameter; MC: Maine Coon; p.A31P: alanine substituted for a proline at residue 31; PW: posterior wall; OR: Odds ratio; SAM: systolic anterior movement; Range: 95% interquartile range.

## Conflict of interest statement

None of the authors of this paper has a financial or personal relationship with other people or organisations that could inappropriately influence or bias the content of the paper

## Authors' contributions

MTNG has designed the study, performed the genetic study and genetic data analysis, performed the statistic data analysis of the clinical data and had the primary responsibility concerning drafting the manuscript. SG and JK both participated in sampling the clinical data and drafting the manuscript, JK also participated in study design. MC participated study design, genetic analysis and drafting the manuscript. All authors read and approved the final manuscript.
